# P004: Implementing an extensive central venous catheter bundle in the Ospedale Papa Giovanni XXIII (formerly Ospedali riuniti) of Bergamo, Italy: experiences and impact

**DOI:** 10.1186/2047-2994-2-S1-P4

**Published:** 2013-06-20

**Authors:** A Raglio, T van der Kooi, G Galbiati, F Averara, A Serna Ortega, E Cucchi, M Ghidini, L Spotti, C Mirabile, A Grigis, S Cesa, M Casati, C Farina, L Lorini, L Chiappa, W Zingg

**Affiliations:** 1 Ospedale Papa Giovanni XXIII, Bergamo, Italy; 2 RIVM, Bilthoven , Netherlands; 3 University hospitals of Geneva, Geneva, Switzerland

## Introduction

As a participant in the PROHIBIT study (Prevention of Hospital Infections by Intervention and Training) our hospital was randomized to evaluate an extensive central venous catheter (CVC) bundle in the intensive care (ICU). Bundle compliance and catheter-related blood stream infections (CRBSI) incidence were monitored.

## Methods

After local approval a team was created with a clinical microbiologist, a study nurse, 3 trainers and 5 ‘champions’ from the ICU team. After a baseline period of 9 months (January-September 2011), a multimodal prevention strategy was initiated including 1) 12 4-hour practical courses for the 260 ICU staff; 2) preparation of a CVC bundle kit; and 3) the update of the written local CVC insertion protocol.

## Results

The baseline CRBSI incidence was 1.0/1000 CVC days (95% confidence interval: 0.5-2.2) and the compliance with all bundle items was 0% (0.0-4.6). A total of 184 out of 1654 CVC insertions was observed. Major bundle limitations were the lack of large sterile drapes (0%), non-observance of skin antisepsis before gowning and sterile draping (9%), and the infrequent use of alcohol-based chlorhexidine for skin antisepsis (27%).

Upon intervention (October 2011 - December 2012) the CRBSI incidence was 1.6 (1.0-2.6). The compliance with all bundle items was never fully achieved (0%, (0.0-2.0). However, this was due to the fact that large drapes could not be purchased due to ICU budget cutbacks.

The compliance of all items including behavioural aspects but excluding the use of large drapes, increased from 80% at baseline to 91% upon intervention.

## Conclusion

Despite the introduction of an extensive CVC bundle in our ICUs, there was no significant difference in CRBSI outcome. The educational part of the introduction seemed successful as the average compliance of many items, including behavioural aspects increased. However, the absence of a reduction shows the limitation of a multimodal intervention strategy in a high performing setting with low CRBSI rates at the outset.

## Disclosure of interest

None declared

